# MOSCHweb — a matrix-based interactive key to the genera of the Palaearctic Tachinidae (Insecta, Diptera)

**DOI:** 10.3897/zookeys.205.3409

**Published:** 2012-07-04

**Authors:** Pierfilippo Cerretti, Hans-Peter Tschorsnig, Massimo Lopresti, Filippo Di Giovanni

**Affiliations:** 1 Dipartimento di Biologia e Biotecnologie “Charles Darwin”, Università di Roma “La Sapienza”, Piazzale A. Moro 5, 00185, Rome, Italy; 2 Centro Nazionale per lo Studio e la Conservazione della Biodiversità Forestale – Corpo Forestale dello Stato, Via Carlo Ederle 16/A, 37100, Verona, Italy; 3 Staatliches Museum für Naturkunde, Rosenstein 1, 70191 Stuttgart, Germany

**Keywords:** Interactive key, identification tool, web application, data matrix, morphology, description protocol, Diptera, Tachinidae, Palaearctic Region

## Abstract

We provide a general overview of features and technical specifications of an original interactive key web application for the identification of Palaearctic Tachinidae genera. The full list of terminal taxa included in the key, which is the most updated list of genera currently recorded for the Palaearctic Region, is given. We also briefly discuss the need for dealing with detailed and standardized taxa descriptions as a base to keep matrix-based interactive tools easily updated, by proposing a standardized protocol.

## Introduction

With more than 1,500 valid genera worldwide (cf. O’Hara 2011), the Diptera family Tachinidae represents a good model to which “alternative” diagnostic tools to the traditional dichotomous keys can be applied. We here propose an original interactive/multi-entry key web application, MOSCHweb (“mosch” derives from the Italian words “mosca” meaning fly and “chiave” meaning key), for the identification of 423 terminal taxa (at generic and subgeneric rank) of Palaearctic Tachinidae (Insecta, Diptera) based on a <characters x taxa> data matrix approach (cf. Dallwitz 1980; Walter and Winterton 2007). The system adopted in MOSCHweb allows the selection of one or more states for each character, while the software discards all taxa that do not share these states; the selection process is repeated until the search is narrowed down to a single taxon.

Unlike traditional dichotomous keys where characters appear in a fixed order and possible difficulties to recognize the state of one or more characters jeopardize the identification process, in MOSCHweb characters have equal value, appear simultaneously, and can be used in any order. This approach allows the user to simply ignore characters of difficult interpretation or characters that are inapplicable due to damage to the specimen. The possibility to proceed in spite of the incompleteness of the specimen is permitted by the high redundancy of the data matrix which is based on a great amount of information about each terminal taxon. Another peculiarity of the key is the possibility for the user to express uncertainty by selecting more than one state per character or even initially selecting all states and then deselecting one state at a time for each character.

At all times, the user is able to keep all the selected characters and states under control. At the end of the identification process, the set of chosen states will form a code associated with each identified specimen.

We also provide dedicated pages for the morphological terminology adopted (including chaetotaxy), the graphic representation of main measurements, and an iconographic database for each character state used in the interactive key. Also included are images and information concerning morphological features and distributions.

Newly described taxa or nomenclatural changes will not alter the logic of the system, as new species can be added as terminal taxa just by adding a row of listed characters for the new species.

### Format of the paper

This paper was prepared following the outlines for data papers provided by Penev et al. (2009, 2011).

## Project description

### Taxonomic coverage

The key covers 414 of the 416 genera belonging to the family Tachinidae that are currently recorded in the Palaearctic Region (cf. Herting 1984; Herting and Dely-Draskovits 1993; Tschorsnig and Richter 1998; Richter 2004; Shima 2006; Cerretti 2010; O’Hara 2011). The terminal taxa of the key are 423 because 8 subgenera and 1 species group were treated separately (see the list below). The following genera are not included in the present version of MOSCHweb because we have not yet examined any specimens: ***Montuosa*** Chao & Zhou, 1996, known from Palaearctic China (cf. O’Hara et al. 2009); ***Aesia*** Richter, 2011, known from the type locality of Wrangel Island (Russia) (Richter 2011).

### List of the terminal taxa included in the current version of the database (last update: April 2012)

***Acemya*** Robineau-Desvoidy, 1830; ***Actia*** Robineau-Desvoidy, 1830; ***Actinochaetopteryx*** Townsend, 1927; ***Adenia*** Robineau-Desvoidy, 1863 [subgenus of *Exorista*]; ***Admontia*** Brauer & Bergenstamm, 1889; ***Allophorocera*** Hendel, 1901; ***Alloprosopaea*** Villeneuve, 1923; ***Alophorophasia*** Townsend, 1927; ***Alsomyia*** Brauer & Bergenstamm, 1891; ***Amelibaea*** Mesnil, 1955; ***Amnonia*** Kugler, 1971; ***Amphicestonia*** Villeneuve, 1939; ***Anaeudora*** Townsend, 1933; ***Ancistrophora*** Schiner, 1865; ***Anechuromyia*** Mesnil & Shima, 1979; ***Aneogmena*** Brauer & Bergenstamm, 1891; ***Anthomyiopsis*** Townsend, 1916; ***Anurophylla*** Villeneuve, 1938; ***Aphantorhaphopsis*** Townsend, 1926 [subgenus of *Siphona*]; ***Aphria*** Robineau-Desvoidy, 1830; ***Aplomya*** Robineau-Desvoidy, 1830; ***Arama*** Richter, 1972; ***Arcona*** Richter, 1988; ***Argyrophylax*** Brauer & Bergenstamm, 1889; ***Athrycia*** Robineau-Desvoidy, 1830; ***Atylomyia*** Brauer, 1898; ***Atylostoma*** Brauer & Bergenstamm, 1889; ***Aulacephala*** Macquart, 1851; ***Austrophorocera*** Townsend, 1916; ***Bactromyia*** Brauer & Bergenstamm, 1891; ***Bampura*** Tschorsnig, 1983; ***Baumhaueria*** Meigen, 1838; ***Belida*** Robineau-Desvoidy, 1863; ***Bessa*** Robineau-Desvoidy, 1863; ***Besseria*** Robineau-Desvoidy, 1830; ***Billaea*** Robineau-Desvoidy, 1830; ***Biomeigenia*** Mesnil, 1961; ***Bithia*** Robineau-Desvoidy, 1863; ***Blepharella*** Macquart, 1951; ***Blepharipa*** Rondani, 1856; ***Blepharomyia*** Brauer & Bergenstamm, 1889; ***Blondelia*** Robineau-Desvoidy, 1830; ***Botria*** Rondani, 1856; ***Brachicheta*** Rondani, 1861; ***Brachymera*** Brauer & Bergenstamm, 1889; ***Bracteola*** Richter, 1972; ***Brullaea*** Robineau-Desvoidy, 1863; ***Buquetia*** Robineau-Desvoidy, 1847; ***Cadurcia*** Villeneuve, 1926; ***Cadurciella*** Villeneuve, 1927; ***Calliethilla*** Shima, 1979; ***Calozenillia*** Townsend, 1927; ***Calyptromyia*** Villeneuve, 1915; ***Campylocheta*** Rondani, 1859; ***Carbonilla*** Mesnil, 1974; ***Carcelia*** Robineau-Desvoidy, 1830; ***Carcelina*** Mesnil, 1944; ***Catagonia*** Brauer & Bergenstamm, 1891; ***Catena*** Richter, 1975; ***Catharosia*** Rondani, 1868; ***Cavalieria*** Villeneuve, 1908; ***Cavillatrix*** Richter, 1986; ***Ceracia*** Rondani, 1865; ***Ceranthia*** Robineau-Desvoidy, 1830 [subgenus of *Siphona*]; ***Ceratochaetops*** Mesnil, 1954; ***Ceromasia*** Rondani, 1856; ***Ceromya*** Robineau-Desvoidy, 1830; ***Cestonia*** Rondani, 1861; ***Cestonionerva*** Villeneuve, 1929; ***Cestonioptera*** Villeneuve, 1939; ***Chaetexorista*** Brauer & Bergenstamm, 1895; ***Chaetoria*** Becker, 1908; ***Chaetovoria*** Villeneuve, 1920; ***Chetina*** Rondani, 1856; ***Chetogena*** Rondani, 1856 [except subgenus *Diplostichus*]; ***Chetoptilia*** Rondani, 1862; ***Chrysomikia*** Mesnil, 1970; ***Chrysosomopsis*** Townsend, 1916; ***Ciala*** Richter, 1976; ***Cinochira*** Zetterstedt, 1845; ***Cistogaster*** Latreille, 1829; ***Clairvillia*** Robineau-Desvoidy, 1830; ***Clairvilliops*** Mesnil, 1959; ***Clausicella*** Rondani, 1856; ***Clemelis*** Robineau-Desvoidy, 1863; ***Cleonice*** Robineau-Desvoidy, 1863; ***Clytiomya*** Rondani, 1861; ***Cnephaotachina*** Brauer & Bergenstamm, 1894 [subgenus of *Nowickia*]; ***Compsilura*** Bouché, 1834; ***Compsiluroides*** Mesnil, 1953; ***Conogaster*** Brauer & Bergenstamm, 1891; ***Conoptina*** Richter, 1995 [subgenus of *Lixophaga*]; ***Corybantia*** Richter, 1986; ***Crapivnicia*** Richter, 1995; ***Crassicornia*** Kugler, 1980; ***Crosskeya*** Shima & Chao, 1988; ***Crypsina*** Brauer & Bergenstamm, 1889; ***Ctenophorinia*** Mesnil, 1963; ***Cucuba*** Richter, 2008; ***Cylindromyia*** Meigen, 1803; ***Cyrtophloeba*** Rondani, 1856; ***Cyzenis*** Robineau-Desvoidy, 1863; ***Datvia*** Richter, 1972; ***Demoticoides*** Mesnil, 1953; ***Demoticus*** Macquart, 1854; ***Dexia*** Meigen, 1826; ***Dexiomimops*** Townsend, 1926; ***Dexiosoma*** Rondani, 1856; ***Dexiotrix*** Villeneuve, 1936; ***Dicarca*** Richter, 1993; ***Dinera*** Robineau-Desvoidy, 1830; ***Dionaea*** Robineau-Desvoidy, 1830; ***Dionomelia*** Kugler, 1978; ***Diplostichus*** Brauer & Bergenstamm, 1889 [subgenus of *Chetogena*]; ***Dolichocolon*** Brauer & Bergenstamm, 1889; ***Dolichocoxys*** Townsend, 1927; ***Dolichopodomintho*** Townsend, 1927; ***Drino*** Robineau-Desvoidy, 1863; ***Drinomyia*** Mesnil, 1962; ***Dufouria*** Robineau-Desvoidy, 1830; ***Ectophasia*** Townsend, 1912; ***Elfriedella*** Mesnil, 1957; ***Eliozeta*** Rondani, 1856; ***Eloceria*** Robineau-Desvoidy, 1863; ***Elodia*** Robineau-Desvoidy, 1863; ***Elomya*** Robineau-Desvoidy, 1830; ***Emporomyia*** Brauer & Bergenstamm, 1891; ***Engeddia*** Kugler, 1977; ***Entomophaga*** Lioy, 1864; ***Epicampocera*** Macquart, 1849; ***Erebiomima*** Mesnil, 1953; ***Eriothrix*** Meigen, 1803; ***Erycesta*** Herting, 1967; ***Erycia*** Robineau-Desvoidy, 1830; ***Erynnia*** Robineau-Desvoidy, 1830; ***Erynniopsis*** Townsend, 1926; ***Erythrocera*** Robineau-Desvoidy, 1848; ***Estheria*** Robineau-Desvoidy, 1830; ***Ethilla*** Robineau-Desvoidy, 1863; ***Etroga*** Richter, 1995; ***Eubrachymera*** Townsend, 1919; ***Euexorista*** Townsend, 1912; ***Eugymnopeza*** Townsend, 1933; ***Euhygia*** Mesnil, 1960; ***Eulabidogaster*** Belanovsky, 1951; ***Eulasiona*** Townsend, 1892; ***Eumea*** Robineau-Desvoidy, 1863; ***Eumeella*** Mesnil, 1939; ***Eurysthaea*** Robineau-Desvoidy, 1863; ***Euthera*** Loew, 1866; ***Eutrixopsis*** Townsend, 1919; ***Euvespivora*** Baranov, 1942; ***Everestiomyia*** Townsend, 1933; ***Exorista*** Meigen, 1803 [except subgenus *Adenia*]; ***Feriola*** Mesnil, 1957; ***Fischeria*** Robineau-Desvoidy, 1830; ***Flavicorniculum*** Chao & Shi, 1981; ***Freraea*** Robineau-Desvoidy, 1830; ***Frontina*** Meigen, 1838; ***Gaedia*** Meigen, 1838; ***Galsania*** Richter, 1993; ***Gastrolepta*** Rondani, 1862; ***Gastroptilops*** Mesnil, 1957; ***Germaria*** Robineau-Desvoidy, 1830; ***Germariochaeta*** Villeneuve, 1937; ***Glaurocara*** Thomson, 1869; ***Gnadochaeta*** Macquart, 1851; ***Gonia*** Meigen, 1803; ***Goniocera*** Brauer & Bergenstamm, 1891; ***Goniophthalmus*** Villeneuve, 1910; ***Graphogaster*** Rondani, 1868; ***Gymnocheta*** Robineau-Desvoidy, 1830; ***Gymnoglossa*** Mik, 1898; ***Gymnomacquartia*** Mesnil & Shima, 1979; ***Gymnophryxe*** Villeneuve, 1922; ***Gymnosoma*** Meigen, 1803; ***Halidaya*** Egger, 1856; ***Hamaxia*** Walker, 1860; ***Hamaxiella*** Mesnil, 1967; ***Hapalioloemus*** Baranov, 1934; ***Haracca*** Richter, 1995; ***Hasmica*** Richter, 1972; ***Hebia*** Robineau-Desvoidy, 1830; ***Hemimacquartia*** Brauer & Bergenstamm, 1893; ***Hemyda*** Robineau-Desvoidy, 1830; ***Heraultia*** Villeneuve, 1920; ***Hermya*** Robineau-Desvoidy, 1830; ***Hubneria*** Robineau-Desvoidy, 1847; ***Hyalurgus*** Brauer & Bergenstamm, 1893; ***Hyleorus*** Aldrich, 1926; ***Hyperaea*** Robineau-Desvoidy, 1863; ***Hypovoria*** Villeneuve, 1912; ***Hystriomyia*** Portshinsky, 1881; ***Imitomyia*** Townsend, 1912; ***Isafarus*** Richter, 1976; ***Isosturmia*** Townsend, 1927; ***Istocheta*** Rondani, 1859; ***Janthinomyia*** Brauer & Bergenstamm, 1893; ***Kallisomyia*** Borisova-Zinov’eva, 1964; ***Kirbya*** Robineau-Desvoidy, 1830; ***Klugia*** Robineau-Desvoidy, 1863; ***Kuwanimyia*** Townsend, 1916; ***Labigastera*** Macquart, 1834; ***Lambrusca*** Richter, 1998; ***Lasiopales*** Villeneuve, 1922; ***Laufferiella*** Villeneuve, 1929; ***Lecanipa*** Rondani, 1859; ***Leiophora*** Robineau-Desvoidy, 1863; ***Leptothelaira*** Mesnil & Shima, 1979; ***Leskia*** Robineau-Desvoidy, 1830; ***Leucostoma*** Meigen, 1803; ***Ligeria*** Robineau-Desvoidy, 1863; ***Ligeriella*** Mesnil, 1961; ***Linnaemya*** Robineau-Desvoidy, 1830; ***Lissoglossa*** Villeneuve, 1912; ***Litophasia*** Girschner, 1887; ***Lixophaga*** Townsend, 1908 [*sensu stricto*]; ***Loewia*** Egger, 1856; ***Lomachantha*** Rondani, 1859; ***Lophosia*** Meigen, 1824; ***Lydella*** Robineau-Desvoidy, 1830; ***Lydina*** Robineau-Desvoidy, 1830; ***Lypha*** Robineau-Desvoidy, 1830; ***Lyphosia*** Mesnil, 1957; ***Macquartia*** Robineau-Desvoidy, 1830; ***Macroprosopa*** Brauer & Bergenstamm, 1889; ***Maculosalia*** Mesnil, 1946; ***Madremyia*** Townsend, 1916; ***Magripa*** Richter, 1988; ***Manola*** Richter, 1982; ***Masicera*** Macquart, 1834; ***Masistyloides*** Mesnil, 1963; ***Masistylum*** Brauer & Bergenstamm, 1893; ***Medina*** Robineau-Desvoidy, 1830; ***Meigenia*** Robineau-Desvoidy, 1830; ***Melisoneura*** Rondani, 1861; ***Mendelssohnia*** Kugler, 1971; ***Mesnilisca*** Zimin, 1974; ***Metacemyia*** Herting, 1969; ***Metadrinomyia*** Shima, 1980; ***Microcerophina*** Kugler, 1977; ***Microphthalma*** Macquart, 1843; ***Microsoma*** Macquart, 1855; ***Mikia*** Kowarz, 1885; ***Milada*** Richter, 1973; ***Mintho*** Robineau-Desvoidy, 1830; ***Minthodes*** Brauer & Bergenstamm, 1889; ***Mitannia*** Herting, 1987; ***Mongolomintho*** Richter, 1976; ***Munira*** Richter, 1974; ***Mycteromyiella*** Mesnil, 1966; ***Myxexoristops*** Townsend, 1911; ***Naira*** Richter, 1970; ***Nanoplagia*** Villeneuve, 1929 [removed from synonymy with *Plagiomima* Brauer & Bergenstamm, 1891] (cf. Cerretti 2009); ***Neaera*** Robineau-Desvoidy, 1830; ***Nealsomyia*** Mesnil, 1939; ***Nemoraea*** Robineau-Desvoidy, 1830; ***Nemorilla*** Rondani, 1856; ***Neoemdenia*** Mesnil, 1953; ***Neophryxe*** Townsend, 1916; ***Neoplectops*** Malloch, 1930; ***Nigara*** Richter, 1999; ***Nilea*** Robineau-Desvoidy, 1863; ***Nipponoceromyia*** Mesnil & Shima, 1978; ***Nowickia*** Wachtl, 1894 [*sensu stricto*]; ***Oblitoneura*** Mesnil, 1975; ***Ocytata*** Gistel, 1848; ***Onychogonia*** Brauer & Bergenstamm, 1889; ***Opesia*** Robineau-Desvoidy, 1863; ***Oswaldia*** Robineau-Desvoidy, 1863; ***Oxyphyllomyia*** Villeneuve, 1937; ***Pachycheta*** Portschinsky, 1881; ***Pachystylum*** Macquart, 1848; ***Pales*** Robineau-Desvoidy, 1830; ***Palesisa*** Villeneuve, 1929; ***Palmonia*** Kugler, 1972; ***Pandelleia*** Villeneuve, 1907; ***Panzeria*** Robineau-Desvoidy, 1830 [including the species formerly ascribed to *Ernestia* Robineau-Desvoidy, 1830, *Fausta* Robineau-Desvoidy, 1830, *Eurithia* Robineau-Desvoidy, 1844 and *Appendicia* Stein, 1924 by Herting (1984), Herting and Dely-Draskovits (1993), Tschorsnig et al. 2004] (*cf*. Cerretti 2010); ***Paracraspedothrix*** Villeneuve, 1919; ***Paradrino*** Mesnil, 1949; ***Paralypha*** Mesnil, 1963; ***Parapexopsis*** Mesnil, 1953; ***Parasetigena*** Brauer & Bergenstamm, 1891; ***Paratrixa*** Brauer & Bergenstamm, 1891; ***Paratryphera*** Brauer & Bergenstamm, 1891; ***Parerigone*** Brauer, 1898; ***Parhamaxia*** Mesnil, 1967; ***Pelamera*** Herting, 1969; ***Pelatachina*** Meade, 1894; ***Peleteria*** Robineau-Desvoidy, 1830; ***Pentatomophaga*** de Meijere, 1917; ***Periarchiclops*** Villeneuve, 1924; ***Peribaea*** Robineau-Desvoidy, 1863; ***Perigymnosoma*** Villeneuve, 1929; ***Periscepsia*** Gistel, 1848 [*sensu stricto*]; ***Petagnia*** Rondani, 1856; ***Peteina*** Meigen, 1838; ***Petinarctia*** Villeneuve, 1928 [subgenus of *Periscepsia*]; ***Pexopsis*** Brauer & Bergenstamm, 1889; ***Phania*** Meigen, 1824; ***Phasia*** Latreille, 1804; ***Phebellia*** Robineau-Desvoidy, 1846; ***Phenicellia*** Robineau-Desvoidy, 1863; ***Phonomyia*** Brauer & Bergenstamm, 1893; ***Phorinia*** Robineau-Desvoidy, 1830; ***Phorocera*** Robineau-Desvoidy, 1830; ***Phorocerosoma*** Townsend, 1927; ***Phryno*** Robineau-Desvoidy, 1830; ***Phryxe*** Robineau-Desvoidy, 1830; ***Phyllomya*** Robineau-Desvoidy, 1830; ***Phytomyptera*** [*partim*] [= *Gwenda* Richter, 1977]; ***Phytomyptera*** Rondani, 1845 [except the species formerly included in *Gwenda* Richter]; ***Phytorophaga*** Bezzi, 1923; ***Picconia*** Robineau-Desvoidy, 1863; ***Platymya*** Robineau-Desvoidy, 1830; ***Plesina*** Meigen, 1838; ***Policheta*** Rondani, 1856; ***Pradocania*** Tschorsnig, 1997; ***Proceromyia*** Mesnil, 1957; ***Prodegeeria*** Brauer & Bergenstamm, 1894; ***Prodemoticus*** Villeneuve, 1919; ***Prooppia*** Townsend, 1926; ***Prosena*** Le Peletier & Serville, 1828; ***Prosethilla*** Herting, 1984; ***Prosopea*** Rondani, 1861; ***Psalidoxena*** Villeneuve, 1941; ***Pseudalsomyia*** Mesnil, 1968; ***Pseudebenia*** Shima, Han & Tachi, 2010; ***Pseudogonia*** Brauer & Bergenstamm, 1889; ***Pseudomintho*** Brauer & Bergenstamm, 1889; ***Pseudopachystylum*** Mik, 1891; ***Pseudoperichaeta*** Brauer & Bergenstamm, 1889; ***Ptesiomyia*** Brauer & Bergenstamm, 1893; ***Ramonda*** Robineau-Desvoidy, 1863 [subgenus of *Periscepsia*]; ***Ramonella*** Kugler, 1980; ***Redtenbacheria*** Schiner, 1861; ***Rhacodinella*** Mesnil, 1968; ***Rhamphina*** Macquart, 1835; ***Rhaphiochaeta*** Brauer & Bergenstamm, 1889; ***Rhinaplomyia*** Mesnil, 1953; ***Rhinomyodes*** Townsend, 1933; ***Rhynchogonia*** Brauer & Bergenstamm, 1893; ***Richteriola*** Mesnil, 1963; ***Riedelia*** Mesnil, 1942; ***Rioteria*** Herting, 1973; ***Robinaldia*** Herting, 1983; ***Rondania*** Robineau-Desvoidy, 1850; ***Rossimyiops*** Mesnil, 1953 [= *Mesnilomyia* Kugler, 1972; = *Persedea* Richter 2001] (cf. Cerretti et al. 2009); ***Sarromyia*** Pokorny, 1893; ***Scaphimyia*** Mesnil, 1953; ***Schembria*** Rondani, 1861; ***Schineria*** Rondani, 1857; ***Scomma*** Richter, 1972; ***Senometopia*** Macquart, 1834; ***Sepseocara*** Richter, 1986; ***Sericozenillia*** Mesnil, 1957; ***Setalunula*** Chao & Yang 1990; ***Simoma*** Aldrich, 1926; ***Siphona*** Meigen, 1803 [*sensu stricto*]; ***Sisyropa*** Brauer & Bergenstamm, 1889; ***Smidtia*** Robineau-Desvoidy, 1830; ***Solieria*** Robineau-Desvoidy, 1848; ***Sonaca*** Richter, 1981; ***Spallanzania*** Robineau-Desvoidy, 1830; ***Stackelbergomyia*** Rohdendorf, 1948; ***Staurochaeta*** Brauer & Bergenstamm, 1889; ***Steleoneura*** Stein, 1924; ***Stomina*** Robineau-Desvoidy, 1830; ***Strongygaster*** Macquart, 1834; ***Sturmia*** Robineau-Desvoidy, 1830; ***Sturmiopsis*** Townsend, 1916; ***Subclytia*** Pandellé, 1894; ***Suensonomyia*** Mesnil, 1953; ***Sumpigaster*** Macquart, 1955; ***Symmorphomyia*** Mesnil & Shima, 1977; ***Synactia*** Villeneuve, 1916; ***Synamphichaeta*** Villeneuve, 1936; ***Tachina*** Meigen, 1803; ***Tachinoestrus*** Portshinsky, 1887; ***Takanoella*** Baranov, 1935; ***Takanomyia*** Mesnil, 1957; ***Tetrigimyia*** Shima & Takahashi, 2011; ***Thecocarcelia*** Townsend, 1933; ***Thelaira*** Robineau-Desvoidy, 1830; ***Thelyconychia*** Brauer & Bergenstamm, 1889; ***Thelymorpha*** Brauer & Bergenstamm, 1889; ***Thelymyia*** Brauer & Bergenstamm, 1891; ***Therobia*** Brauer, 1862; ***Thrixion*** Brauer & Bergenstamm, 1889; ***Tlephusa*** Robineau-Desvoidy, 1863; ***Torocca*** Walker, 1859; ***Townsendiellomyia*** Baranov, 1932; ***Trafoia*** Brauer & Bergenstamm, 1893; ***Triarthria*** Stephens, 1829; ***Trichactia*** Stein, 1924; ***Trichoformosomyia*** Baranov, 1934; ***Trichopoda*** Berthold, 1827; ***Trigonospila*** Pokorny, 1886; ***Tritaxys*** Macquart, 1847; ***Trixa*** Meigen, 1824; ***Trixella*** Mesnil, 1980; ***Trixiceps*** Villeneuve, 1936; ***Tryphera*** Meigen, 1838; ***Uclesia*** Girschner, 1901; ***Urodexia*** Osten-Sacken, 1882; ***Uromedina*** Townsend, 1926; ***Ventoplagia*** Richter, 2009; ***Vibrissina*** Rondani, 1861; ***Villanovia*** Strobl, 1910; ***Voria*** Robineau-Desvoidy, 1830; ***Wagneria*** Robineau-Desvoidy, 1830; ***Wardarina*** Mesnil, 1953; ***Weberia*** Robineau-Desvoidy, 1830; ***Weingaertneriella*** Baranov, 1932; ***Winthemia*** Robineau-Desvoidy, 1830; ***Xylotachina*** Brauer & Bergenstamm, 1891; ***Xysta*** Meigen, 1824; ***Zaira*** Robineau-Desvoidy, 1830; ***Zambesomima*** Mesnil, 1967; ***Zenillia*** Robineau-Desvoidy, 1830; ***Zeuxia*** Meigen, 1826; ***Ziminia*** Mesnil, 1963; ***Zophomyia*** Macquart, 1835.

## Characters used in the key

### General features

The key matrix is based on 98 morphological characters of the adult fly. These are encoded into a variable number of character states from 2 to 9, for a total of 374 states. The characters were chosen on externally visible features, accessible even to non-expert users without dissecting the specimens. Male and female terminalia (with the exception of peculiar piercing structures present in females of some genera, clearly visible without special preparation of the specimen) were excluded from this version. The characters used are divided into seven sections (head, antenna, mouthparts, thorax, wing, legs, abdomen), with the easiest and most selective characters being highlighted in green. The user can also enter the length of the specimen in a dedicated box to exclude genera outside the length of the specimen under examination. Moreover, the key allows the user to sort out genera by their subfamily placement or by their parasitized host group affiliation.

### List of the characters used in the key

HEAD: eye pubescence; ocelli; width of frons (male); width of frons (female); outer vertical setae (male); outer vertical setae (female); length of ocellar setae; inclination of ocellar setae; frontal setae; fronto-orbital plate; upper reclinate orbital setae; proclinate orbital setae (male); proclinate orbital setae (female); parafacial setae; width of parafacial; parafacial ratio; shape of facial ridge (head in lateral view); setae of facial ridge; type of setae on facial ridge; vibrissa; face; lower facial margin; genal dilation; ventral part of occiput; dorsal part of occiput; height of gena.

ANTENNAE: colour of antennal pedicel; length of antenna; length of first flagellomere; apex of first flagellomere; pubescence of arista; thickness of arista; length of first aristomere; lenght of second aristomere.

MOUTHPARTS: length of prementum; labella; colour of palpus; size and shape of palpus.

THORAX: presutural dark vittae of scutum; prosternal setulae; shape of prosternum; proepisternum; postpronotum; presutural acrostichal setae; presutural dorsocentral setae; postsutural dorsocentral setae; postsutural intra-alar setae; supra-alar setae; katepisternal setae; katepimeron; anepimeral seta; colour of scutellum; number of marginal setae of scutellum; lateral setae of scutellum; length of subapical setae of scutellum; apical scutellar setae; preapical scutellar setae; anatergite; posterior spiracle; postmetacoxal area.

WING: position of lower calypter; marginal shape of lower calypter; setulae on lower calypter surface; wing colour; tegula colour; basicosta colour; second costal segment; costal spine; vein R_1_; basal seta on vein R_4+5_; setae on vein R_4+5_; vein CuA_1_; bend of vein M; stub and prolongation of vein M; fourth costal sector (CS_4_); ratios of sections of vein M; crossvein DM-Cu; petiole.

LEGS: colour of legs; fore coxa; fore tibia; mid tibia; number of preapical setae on hind tibia; length of preapical setae on hind tibia; anterodorsal setae on hind tibia; hind coxa.

ABDOMEN: ground colour of abdomen; abdominal microtomentum (pattern); fusion of abdominal tergites; mid-dorsal depression on syntergite 1+2; marginal setae on syntergite 1+2; median marginal setae on tergites 3 and 4; median discal setae on tergites 3 and 4; tergite 5 length; sternite 4; male abdominal patches of setulae (pattern); female ovipositor.

COLOUR: general body ground colour.

## Software technical specification

*Platform*: Framework.Net

*Web Server*: Microsoft Internet Information Service 6.0

*Programming language*: C#

*Application version*: MOSCHweb 1.0

*Data base*: Microsoft SQL Server

*Data*: 1.0beta

*Language*: English

*License for use of the key*:Creative Commons Attribution License 3.0 (CC-BY), which permits unrestricted use, distribution, and reproduction in any medium, provided the original author and source are credited.

*Use of the primary data*: Primary data are available from the authors by agreement.

*Web Location*: www.tachinidae.eu

### Software technical features (Fig. 1)

**Figure 1.**
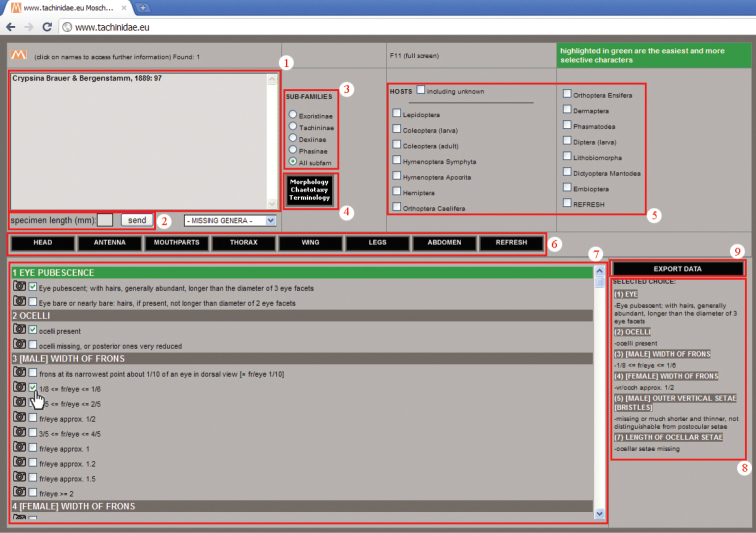
Interactive key main page.

1.* Genera window*: an updating real-time box containing all the genera that share selected character states. The name of the genus is followed by the author name, the year and page of the original description paper. Clicking on the genus name, a new window opens to show the general distribution, body length range and general remarks of the genus, the name of the subfamily it belongs to and the images available for it.

2. *Specimen length box*: a small box in which user can insert an approximate specimen length as an integer number expressed in millimeters.

3.* Subfamilies menu*: a menu giving the possibility to reduce the query to the taxa belonging to just one of the four subfamilies or, by default, to work with the entire data set.

**Figure 2.**
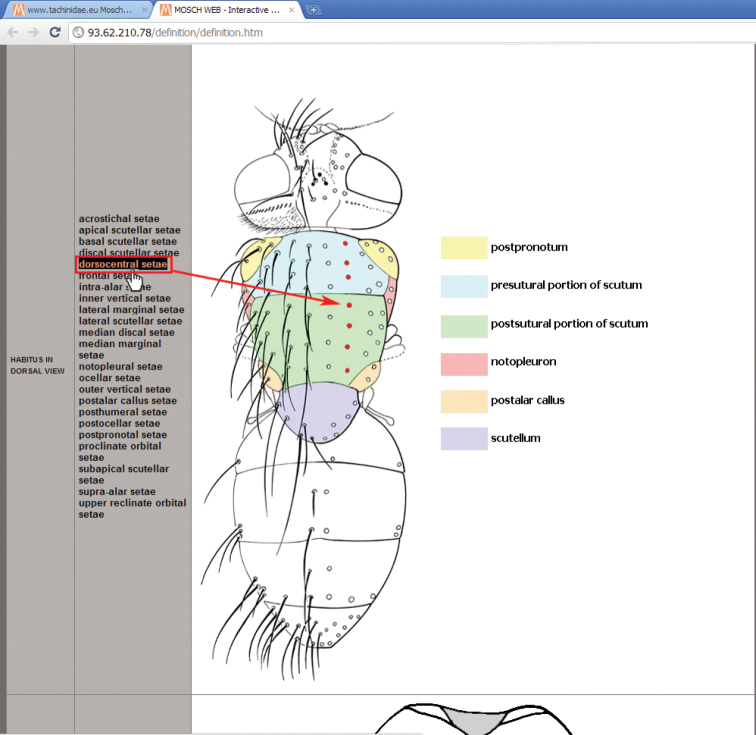
Interactive image window for morphology, chaetotaxy and terminology.

**Figure 3.**
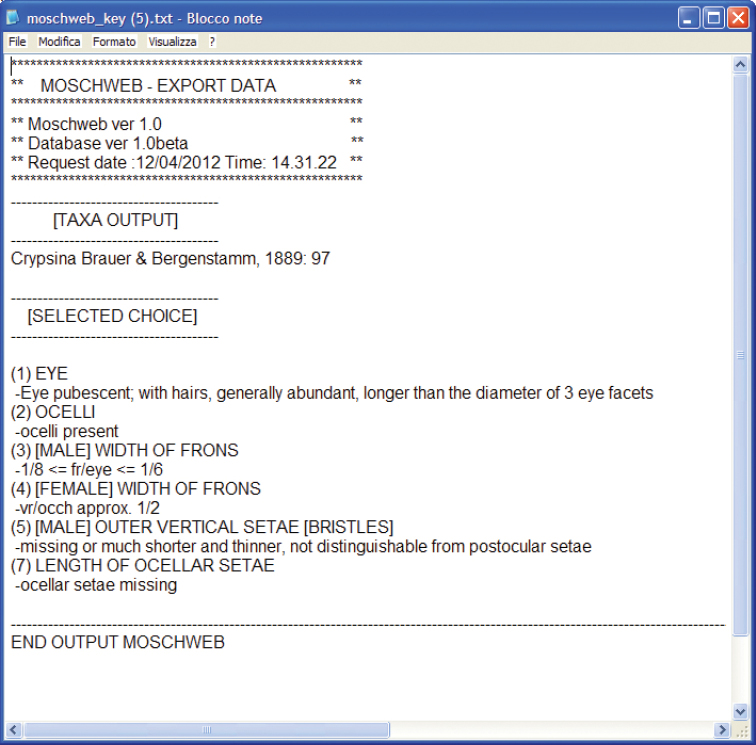
Example of TXT export data file.

4. *Morphology—Chaetotaxy—Terminology button*: a button that refers to a dedicated window (Fig. 2), illustrating the characters used in the key, with the help of interactive images of the body parts, obtainable just by moving the mouse over the list of terms.

5. *Host menu*: a menu that allows data on host relationships to be used as a selection criterion, e.g. with reared specimens. It is possible to include also genera for which host relationships are still unknown. A “refresh” button clears the checkboxes for host (without refreshing the character or subfamily selection).

6. *Body parts bar*: a bar with buttons referring to the body sections where used characters are divided. A “refresh” button clears the checkboxes for characters (without refreshing the host or subfamily selection).

7. *Character window*: a window with all the characters used in the key. Each character has from two to nine states; for every state the user can see the pictures in the archive that refer to that state just by clicking on the camera icon to the left of the checkboxes. The characters can be used in any order; easiest and more selective characters are highlighted in green. MOSCHweb allows also for “uncertainty” to be expressed by the selection of more than one state for each character, as is useful for qualitative or morphometric characters. Taking into account that morphometric ratios are often continuous, we chose to subdivide arbitrarily such characters in more or less regular intervals. For specimens showing values at the extremes of the intervals, it is possible and suggested to select both the states with contiguous values.

8. *Selected choice box*: an updating real-time box showing the chosen characters and states selected by the user, ordered as they appear in the Character window; this represents an ID code which is linked to the specimen under examination.

9. *Export data*: a button allowing the user to export in TXT format the terminal taxon/taxa name (depending upon the accuracy of the inquiry) followed by the list of selected states (point 8) in the form of a code (Fig. 3). This “code” serves as a record of the character states used to achieve a specimen identification. This functionality may be useful to check previous identifications in the light of nomenclatorial changes, group revisions or new taxon descriptions.

MOSCHweb is a user friendly application based on an intuitive graphical interface and very simple dynamics, designed to meet the needs of both specialist and less experienced users. MOSCHweb does not constrain nor address the user to a path in character choice except for highlighting in green those characters that allow a nearly linear reduction of terminal taxa. We think this is a strength of our application. As a consequence, we deliberately excluded both the possibility of removing automatically the redundant characters or states during the identification process and resorting to a probabilistic identification by implementing error tolerance in chosen character states, as it is present in other widely used software packages (cf. Dallwitz 2000 onwards).

### Software implementation and data matrix updating

MOSCHweb is an open-access web application, it is not open-source. The application can be augmented/updated only by, or in agreement with, the corresponding authors of this paper.

Instead, the author of a new taxon is encouraged to download the form of the encoded description from www.tachinidae.eu, fill it out, and send it to the corresponding authors of this paper along with the PDF version of the original description. The author/s of the new taxon/taxa can also attach, to the e-mail, all relevant digital images (e.g., habitus, body parts) to be uploaded to the web application, along with a statement declaring that the images are original or copyright free.

Corresponding authors (PC, HPT) keep updated both the web application, by implementing new functions, and the data matrix, by improving encoded descriptions of terminal taxa. Every change can be monitored on the homepage and reported in the TXT export data file, by updating the number of the application version and by changing the date of the last modification to the data matrix. A short message on the homepage may describe differences from the previous version, if needed.

## Conclusions

It is well known how the high rate of description of new taxa and the many nomenclatural changes (especially among insects) quickly make conventional dichotomous keys obsolete. It is not always easy to update a dichotomous key especially when one or more taxa are split and new “couplets” are needed. Generally, taxonomic and nomenclatorial changes are not a big problem for specialists, but for beginners or general users the only solution is to wait (often in vain) for the publication of a new updated key. In MOSCHweb this problem can be easily solved by augmenting the database (taxa x characters) with the new taxon/taxa. In this way the newly inserted entities will not alter the logic of the system, and automatically become part of the interactive key.

An online open-access resource like MOSCHweb may enhance taxonomic reliability in two ways:

i) By being easily updated once a new genus (or subgenus) is described and published. To do this, taxonomists may simply follow the same strict protocol for taxa description as used in MOSCHweb as a base. The mandatory fields of the description protocol would represent the minimum amount of information recommended for describing a new taxon.

ii) By allowing the recording of the character states selected to identify a given specimen in the form of a TXT file, as a record for the user of the key and for the recipient of the identification.

MOSCHweb, although originally conceived for the identification of a difficult group of parasitoids like the Tachinidae, is to be considered a platform to use also with other taxonomic groups. Those interested in testing MOSCHweb with other groups can send us a list of terminal taxa, a list of characters and character states and the resulting <characters x taxa> matrix. Moreover, we strongly encourage to provide images of treated taxa and pictures referring to the character states present in the key, as well as images illustrating morphological terminology adopted.
